# Initial state of single spines affects probability of induction of cerebellar long term depression in a stochastic model

**DOI:** 10.1186/1471-2202-14-S1-P92

**Published:** 2013-07-08

**Authors:** Anant Jain, Iain Hepburn, Weiliang Chen, Erik De Schutter

**Affiliations:** 1 Computational Neuroscience Unit, Okinawa Institute of Science and Technology, Okinawa, Japan; 2 Theoretical Neurobiology, University of Antwerp, B-2610 Antwerpen, Belgium

## 

Cerebellar long-term depression (LTD) is a type of synaptic plasticity which shows a robust and persistent decrease in the synaptic transmission between parallel fibers (PF) and Purkinje cells (PC), expressed as a reduction in the number of synaptic AMPA receptors. Our lab recently developed a stochastic model of the LTD signaling network, including a PKC-ERK-cPLA_2 _positive feedback loop and mechanisms of AMPAR trafficking, and tuned the model to replicate calcium uncaging experiments [1]. Probabilistically, signaling activity switches between two discrete stable states (LTD or non-LTD). In single spines, the probability of LTD occurrence is only modulated by the concentration and duration of the signal used to trigger it, and inputs with the same magnitude give rise to one of two responses.

The detailed kinetic model of the signaling and trafficking network involved in the induction of cerebellar LTD was developed based on extensive experimental data. The model was solved stochastically and deterministically using STEPS (http://steps.sourceforge.net/), a well validated simulator that implements the Stochastic Simulation Algorithm (SSA)[2].

The reasons for the bistable behavior in single spines is not fully understood. Molecular memory of the compartment, i.e. initial state of molecules before calcium injection, may play a role in altering the probability of a spine to undergo LTD induction or not. We address the possible effects of this initial state on the observed bistable behavior quantitatively. Preliminary analysis of these initial conditions for several simulations lead to their categorization on the basis of LTD probability observed for a particular calcium concentration and pulse duration (for simplicity we named them as High LTD likelihood, Normal LTD likelihood and Low LTD likelihood). We then confirmed these categories for different calcium conditions. The probability of LTD induction has a dependence on calcium pulse duration well-described by the Hill equation. The High and Normal LTD likelihood cases showed similar characteristics, though the Low LTD likelihood category showed different Hill parameters and also didn't saturate at 100 percent. Observations when varying the time before calcium input showed that the High and Normal LTD likelihood states exist only on the order of minutes and dissipate afterwards. In depth understanding of these different initial states may give better insights into the underlying molecular mechanisms causing bistable behavior. This is an important phenomena, as it is also observed in other forms of long-term synaptic plasticity and in many other biological processes.
